# Low-dose aspirin and breast cancer risk: results by tumour characteristics from a randomised trial

**DOI:** 10.1038/sj.bjc.6604240

**Published:** 2008-02-12

**Authors:** S M Zhang, N R Cook, J E Manson, I-M Lee, J E Buring

**Affiliations:** 1 Division of Preventive Medicine, Department of Medicine, Brigham and Women's Hospital, Harvard Medical School, Boston, MA, USA; 2 Department of Epidemiology, Harvard School of Public Health, Boston, MA, USA; 3 Channing Laboratory, Department of Medicine, Brigham and Women's Hospital, Harvard Medical School, Boston, MA, USA; 4 Department of Ambulatory Care and Prevention, Harvard Medical School, Boston, MA, USA

**Keywords:** aspirin, breast cancer, incidence

## Abstract

The Women's Health Study trial previously reported no overall effect of low-dose aspirin (100 mg every other day) on invasive breast cancer over an average of 10 years of treatment. The present subgroup analyses further show no effects by tumour characteristics at diagnosis, suggesting that low-dose aspirin has no preventive effect on breast cancer.

Aspirin inhibits cyclooxygenase enzymes (COX-1 and -2) (DuBois, 2004). Cyclooxygenase 2 (COX-2) overexpression induces the occurrence of mammary tumours in transgenic mice (Liu *et al*, 2001). Prostaglandin E2 that is generated from COX-2 overexpression stimulates the expression of cytochrome *P*450 aromatase, a key enzyme in local oestrogen production, and induces angiogenesis (DuBois, 2004). Cyclooxygenase 2 overexpression occurs in approximately 40% of invasive breast cancer, and is more common in tumours with large size, lymph node metastasis, a ductal type of histology, high histological grade, or negative hormone receptor status (Ristimaki *et al*, 2002). Thus, the effect of aspirin may be stronger in these subtypes of tumours.

In July 2005, the Women's Health Study (WHS) reported overall results from the only randomised trial of aspirin and cancer risk in women (Cook *et al*, 2005). After an average of 10 years of treatment and follow-up, low-dose aspirin (100 mg every other day) had no effect on risk of invasive breast cancer overall or by combined hormone receptor status in 39 876 women aged ⩾45 years. In a subgroup analysis, we evaluate whether low-dose aspirin might reduce risk according to tumour characteristics at diagnosis.

## MATERIALS AND METHODS

### Study design

During 1992–1996, a total of 39 876 women with no history of cancer or cardiovascular disease were enrolled and randomised into a 2 × 2 factorial design of low-dose aspirin (100 mg every other day, provided by the Bayer HealthCare, Leverkusen, Germany) and vitamin E (600 IU every other day, provided by the Natural Source Vitamin E Association) for the primary prevention of cancer and cardiovascular disease (Clinicaltrials.gov identifier, NCT00000479). The methods of the study design have been described in detail previously (Cook *et al*, 2005). Written informed consent was obtained from each participant. The trial was approved by the Human Subjects Committee at the Brigham and Women's Hospital and monitored by an external Data and Safety Monitoring Board.

Annually, participants were sent monthly calendar packs containing study medications, and questionnaires inquiring about potential adverse effects, adherence to pill taking, and occurrence of disease outcomes. Study medications and disease ascertainment were continued in blinded fashion through the scheduled end of the trial (31 March 2004). Deaths of participants were identified by reports from family members, postal authorities, and a search of the National Death Index. Morbidity and mortality follow-up were 97.2 and 99.4% complete, respectively (Cook *et al*, 2005).

For reported diagnoses of breast cancer, medical records and other relevant information were sought and reviewed by physicians who were blinded to randomised treatment assignment for final confirmation. Tumour characteristics at diagnosis were also recorded from medical records. Only confirmed breast cancer cases were included in the analysis. Women who had *in situ* breast cancer and were diagnosed with a new invasive breast cancer at a later date (13 cases) were counted as events in both analyses of *in situ* and invasive cancers.

### Statistical analysis

Analyses used the intent-to-treat principle. The hazard ratios (HRs) and 95% confidence intervals (CIs) of breast cancer comparing women randomised to low-dose aspirin *vs* placebo according to tumour characteristics were computed by Cox proportional hazards regression models with adjustments for age (in years) and randomised treatment assignments of vitamin E (vitamin E *vs* placebo) and *β*-carotene (*β*-carotene *vs* placebo). The proportionality assumption was tested by including an interaction term of aspirin with the logarithm of time and was not violated for total (*P*-value=0.78), invasive (*P*-value=0.87), or *in situ* breast cancer (*P*-value=0.27).

SAS version 9.1 (SAS Institute, Cary, NC, USA) was used for all analyses. All *P*-values were two-sided at the significance level of ⩽0.05.

## RESULTS

Low-dose aspirin treatment had no significant effect on risk of total (762 *vs* 779 cases, HR=0. 98, 95% CI: 0.88–1.08), invasive (608 *vs* 622 cases, HR=0.98, 95% CI: 0.87–1.09) (Cook *et al*, 2005), or *in situ* breast cancers (159 *vs* 165 cases, HR=0.96, 95% CI: 0.78–1.20). Similarly, there were no significant effects of low-dose aspirin on risk of invasive breast cancer according to tumour size, histology, or histologic grading and differentiation (Table 1). However, there appeared a borderline significant increase in risk for tumours with unknown size or metastasised to lymph nodes. In addition, there was no significant effect of low-dose aspirin according to either oestrogen receptor (ER) or progesterone receptor (PR) status (Table 1), or the combined ER and PR status (Cook *et al*, 2005).

## DISCUSSION

In this large randomised trial, an average of 10 years of treatment with low-dose aspirin (100 mg on alternate days) did not affect breast cancer risk overall or by tumour characteristics at diagnosis. The results from the few studies by breast tumour characteristics are conflicting. In a case–control study, aspirin use was associated with a decreased risk for hormone receptor-positive tumours, but not for hormone receptor-negative tumours (Terry *et al*, 2004). By contrast, in the California Teachers Study cohort, daily long-term use of aspirin was not associated with risk for ER+/PR+ breast cancer, but with a significantly increased risk for ER−/PR− breast cancer (Marshall *et al*, 2005). Also, no association was observed for either localised or unlocalised breast tumours. In the Multiethnic Cohort, duration of aspirin use was not associated with risk of breast tumours positive for ER and/or PR or negative for both (Gill *et al*, 2007). In the WHS trial, besides a lack of effect according to ER and PR status, there were no significant effects of low-dose aspirin by tumour size, lymph node metastasis, histology, or histologic grading and differentiation. The borderline significant results for tumours with unknown size or metastasised to lymph nodes were likely a result of chance. Taken together, the current data do not provide evidence for an association of aspirin use with risk of breast cancer by tumour characteristics.

Strengths of this study include a randomised, double-blind, placebo-controlled design, which minimises the confounding and biases that potentially affect observational studies, and the results cannot readily be explained by inadequate duration of treatment and follow-up.

The possibility remains that our lack of effect of aspirin might be due to inadequate dose. However, in colorectal tissues, daily 40.5, 81, 325, and 650 mg doses of aspirin have a similar inhibitory effect on the production of prostaglandins (Ruffin *et al*, 1997). The results from observational studies by aspirin doses are far from consistent. In the Women's Health Initiative cohort, only regular-dose (325 mg) aspirin, but not low-dose (81 mg) aspirin, was associated with a reduced risk of breast cancer (Harris *et al*, 2003). In a study using a Canadian automated database, there was no association for frequent use of aspirin at a dose of ⩽100 mg day^−1^, but an inverse association at a dose of >100 mg day^−1^ (Rahme *et al*, 2005). By contrast, in the UK General Practice Research Database, a significant reduction was seen only for a daily 75 mg dose, but not for daily doses of 150 and 300 mg (Garcia Rodriguez and Gonzalez-Perez, 2004). Furthermore, the Vitamins and Lifestyle Study reported a significantly reduced risk with the use of low-dose aspirin at ⩾4 days week^−1^ over 10 years, whereas an increased risk with frequent use of regular or extra strength aspirin (Ready *et al*, 2007).

In summary, findings from this large randomised trial do not provide support for the use of low-dose aspirin as a chemopreventive agent for breast cancer.

## Figures and Tables

**Table 1:** Hazard ratios of invasive breast cancer according to randomised aspirin treatment, by tumour characteristics in the Women's Health Study

	**Aspirin**	**Placebo**	**Hazard ratio**	
**Variable**	**(*n*=19 934)**	**(*n*=19 942)**	**(95% CI)**	***P*-value**
Invasive breast cancer – no. of cases	608	622	0.98 (0.87, 1.09)	0.68
				
*Tumour size*				
⩽2 cm	436	457	0.95 (0.84, 1.09)	0.48
>2–5 cm	129	126	1.02 (0.80, 1.31)	0.86
>5 cm	12	18	0.67 (0.32, 1.39)	0.28
Any size with direct extension to chest wall or skin	2	4	0.50 (0.09, 2.73)	0.42
Missing	29	17	1.71 (0.94, 3.10)	0.08
				
*Lymph nodes*				
No metastasis	415	453	0.92 (0.80, 1.05)	0.19
Metastasis to lymph nodes	163	134	1.22 (0.97, 1.53)	0.09
Missing	30	35	0.86 (0.53, 1.39)	0.53
				
*Histology*				
Duct carcinoma	438	452	0.97 (0.85, 1.10)	0.63
Lobular carcinoma	64	76	0.84 (0.60, 1.17)	0.31
Duct and lobular carcinoma	54	45	1.20 (0.81, 1.78)	0.37
Adenocarcinoma	3	5	0.60 (0.14, 2.52)	0.49
Tubular adenocarcinoma	13	19	0.68 (0.34, 1.39)	0.29
Mucinous adenocarcinoma	14	11	1.27 (0.58, 2.79)	0.56
Medullary carcinoma	4	4	1.00 (0.25, 4.00)	>0.99
Other	18	10	1.80 (0.83, 3.90)	0.14
				
*Histologic grading and differentiation*				
Well differentiated	127	147	0.86 (0.68, 1.10)	0.22
Moderately differentiated	249	253	0.98 (0.83, 1.17)	0.85
Poorly differentiated/anaplastic	150	143	1.05 (0.83, 1.32)	0.68
Missing	82	79	1.04 (0.76, 1.41)	0.82
				
*Oestrogen receptor status*				
Positive	473	496	0.95 (0.84, 1.08)	0.45
Negative	101	100	1.01 (0.77, 1.33)	0.94
Borderline	5	1	5.00 (0.59, 42.8)	0.14
Missing	29	25	1.16 (0.68, 1.98)	0.59
				
*Progesterone receptor status*				
Positive	421	428	0.98 (0.86, 1.12)	0.80
Negative	147	155	0.95 (0.76, 1.19)	0.65
Borderline	5	3	1.66 (0.40, 6.96)	0.49
Missing	35	36	0.97 (0.61, 1.55)	0.91

CI, confidence interval.
